# ID 235 - Monitoramento dos Prazos de Medicamentos Incorporados no Âmbito do SUS

**DOI:** 10.5327/2237-9622.2025.v34s1.235

**Published:** 2025-11-25

**Authors:** Samara Helena de Carvalho, Amanda Oliveira Lyrio, Ana Carolina de Freitas Lopes, Luciene Fontes Schluckebier Bonan

**Keywords:** implementação, monitoramento pós-incorporação, medicamentos

## Abstract

**Introdução:**

O monitoramento do tempo de implementação de tecnologias em saúde é crucial para garantir o acesso rápido a medicamentos considerados custo-efetivos pela Comissão Nacional de Incorporação de Tecnologias no SUS (Conitec). No Brasil, o prazo legal para implementação é de 180 dias após a publicação da portaria de incorporação. A demora nesse processo pode prejudicar a saúde da população, retardando o acesso a tratamentos essenciais. Este estudo visa analisar os intervalos entre a incorporação e a disponibilização de medicamentos no Sistema Único de Saúde (SUS), destacando o tempo necessário para atingir os principais marcos do processo de implementação.

**Método:**

Foram selecionados medicamentos do Componente Especializado da Assistência Farmacêutica (Ceaf), recomendados pela Conitec em 2019 e 2020, para incorporação ou ampliação de uso. Apenas os medicamentos dos Grupos 1A e 1B foram incluídos, devido à melhor qualidade dos registros de utilização. Medicamentos que não haviam sido implementados até agosto de 2024 ou que não apresentavam dados disponíveis de utilização foram excluídos. Foram avaliados marcos importantes do processo, como a pactuação de financiamento na Comissão Intergestores Tripartite (CIT); a publicação da portaria do Protocolo Clínico e Diretrizes Terapêuticas (PCDT); a criação ou atualização do Sistema de Gerenciamento da Tabela de Procedimentos, Medicamentos e Órteses, Próteses e Materiais Especiais do SUS (Sigtap); e a implementação, definida pelo primeiro registro de dispensação no Sistema de Informações Laboratoriais (SIA) para incorporações ou pela atualização do PCDT para ampliações de uso. As datas de pactuação foram extraídas das atas e gravações da CIT, enquanto as demais datas foram obtidas das portarias. A análise considerou a mediana da diferença entre essas datas e a data da portaria de incorporação.

**Resultados:**

Foram incluídos 17 medicamentos, majoritariamente pertencentes ao Grupo 1A do Ceaf (76%). A mediana do tempo para pactuação de financiamento foi de 128 dias (mínimo: 50; máximo: 496), com três medicamentos sem registro de pactuação. Para a publicação do PCDT, a mediana foi de 363 dias (mínimo: 161; máximo: 798). A criação do Sigtap apresentou mediana de 545 dias (mínimo: 180; máximo: 793). A implementação efetiva ocorreu em uma mediana de 562 dias (mínimo: 191; máximo: 932).

**Conclusão:**

O estudo mostrou que, apesar do prazo legal de 180 dias para a implementação de medicamentos incorporados, a maioria dos marcos essenciais para sua disponibilização no SUS ultrapassa esse limite. A pactuação de financiamento foi o único processo que, em grande parte, respeitou o prazo, embora não tenha sido possível identificar a data de pactuação para três medicamentos, ressaltando a necessidade de maior transparência nessas informações. Em contrapartida, os atrasos identificados apontam que há barreiras institucionais e operacionais que comprometem a agilidade no acesso aos medicamentos. O monitoramento pós-incorporação é, portanto, uma ferramenta crítica para identificar esses gargalos e orientar ajustes nas políticas públicas, garantindo que o acesso a medicamentos ocorra de forma mais rápida e eficiente. Ademais, este estudo destaca a importância de uma articulação mais eficaz entre os diversos atores envolvidos no processo de implementação, visando otimizar a gestão de tempo e assegurar o cumprimento dos prazos legais, com o objetivo de promover maior equidade no acesso à saúde.

